# Hyperthyroidism and hepatic dysfunction: Report of 17 cases

**DOI:** 10.1002/jgh3.12337

**Published:** 2020-04-11

**Authors:** Benothman Wafa, Hadjkacem Faten, Elleuch Mouna, Mnif Fatma, Abid Mohamed

**Affiliations:** ^1^ Department of Endocrinology Hedi Chaker Hospital Sfax Tunisia

**Keywords:** cholestasis, hyperthyroidism, liver function test

## Abstract

**Aims:**

Hyperthyroidism has been known to be associated with abnormalities of serum liver chemistry. The objective of our study is to describe clinical, biochemical and therapeutic features of hepatic dysfunction in hyperthyroidism.

**Methods and Results:**

This retrospective study was conducted on patients hospitalized in our endocrinology department over 20 years. We included patients with untreated and noniatrogenic hyperthyroidism among whom biochemical findings noted hepatic dysfunction and excluded those with concomitant liver disease. Our population is composed of 10 men and 7 women. The average age was 41.4 years. The mean serum level of free thyroxine was 83.8 pmol/L. The serum thyrotropin level was below the detection limit in 10/17 cases. Graves' disease was the most frequently found etiology of hyperthyroidism. Fourteen patients had hyperthyroidism's complications. Eleven patients manifested congestive heart failure. Hepatic dysfunction was moderate and severe in eight and two cases, respectively. Fifteen patients had cholestasis, associated with jaundice in five cases. Hepatocellular injury and synthetic liver dysfunction were noted in seven and five cases, respectively. Thyroid peroxidase antibodies were positively correlated with the serum level of bilirubin (*ρ* = 0.695; *P* = 0.038). A negative correlation was noted between alanine aminotransferase and left ventricular ejection fraction (*ρ* = −0.812; *P* = 0.05). Radioactive iodine was indicated in 15/17 cases. Follow‐up liver tests were performed in 11 cases. They all had normalized hepatic function once euthyroidism restored.

**Conclusion:**

Liver injury in hyperthyroidism is relatively common, ranging from mild to severe. Therefore, patients presenting unexplained hepatic abnormalities require close examination and an evaluation of the thyroid function should be sought.

## Graphical abstract

We report on 17 cases of patients hospitalized for hyperthyroidism and hepatic dysfunction. Liver injury in hyperthyroidism is relatively common, ranging from mild to Severe. Therefore, patients presenting unexplained hepatic abnormalities require close examination and an evaluation of the thyroid function should be sought.

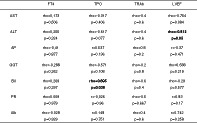

## Introduction

Thyroid disease is a common condition. Excess or deficiency of thyroid hormone is known to have wide‐ranging effects on a variety of organ systems.^1^ Hyperthyroidism is confirmed by subnormal (usually undetectable) serum thyrotropin (TSH) with elevated serum levels of free thyroxine (FT4) as defined by American Thyroid Association.^2^ The three most common causes of thyroid hyperfunction are Graves' disease, multinodular toxic goiter, and autonomous hyperfunctioning thyroid nodule.^3^, ^4^ Clinical presentation of hyperthyroidism is usually mild or moderate. The most severe forms of hyperthyroidism are very rare and, sometimes, accompanied by high morbidity or even mortality.^5^

Hyperthyroidism has been known for the past 70 years to be associated with abnormalities of serum liver chemistry and histology.^6^, ^7^ It has been first reported by Habershon^8^ in 1874.

The objective of our study is to describe clinical, biochemical, and therapeutic features of hepatic dysfunction in hyperthyroidism.

## Methods

This retrospective study included 17 patients, 18 years of age or older, hospitalized in our endocrinology department. Our study was conducted over 20 years, between 1997 and 2017.

We included patients with untreated and noniatrogenic hyperthyroidism in whom biochemical findings noted hepatic dysfunction.

We excluded patients:

- With amiodarone‐induced thyrotoxicosis;
- Previously or currently on antithyroid therapy or any other medications (statins, azole antifungals, isoniazid, valproic acid) that may affect liver and thyroid function tests;
- With a concomitant liver disease.

None of the included patients had diagnoses of autoimmune hepatitis, primary sclerosing cholangitis, or primary biliary cirrhosis. All patients had negative serological markers. Moreover, serologic testing excluded the usual causes of viral hepatitis.

Data were extracted from patient records, including serum thyroid indices and liver function test (LFTs): serum aspartate transaminotransferase (AST), alanine aminotransferase (ALT), alkaline phosphatase (AP), gamma‐glutamyltransferase (GGT), and total bilirubin (bil). None of the patients had isoenzyme analysis to differentiate hepatic from bone AP. Markers of hepatic synthetic function were also assessed, serum albumin (Alb) and prothrombin ratio (PR) in particular. Abdominal ultrasound, computed tomography scan, and echocardiogram were recorded when available.

The diagnosis of hepatic dysfunction was based on the following criteria^9^:

- ALT, AST or GGT < 3 times the upper limit of normal (ULN), AP < 2 ULN, and/or bil < 2.5 ULN was defined as mild hepatic dysfunction;
- 3 ULN≤ALT or AST < 20 ULN, 3 ULN≤GGT < 10 ULN, 2 ULN ≤ AP < 5 ULN, and/or 2.5 ULN≤bil < 5 ULN as moderate;
- ALT or AST ≥ 20 ULN, GGT ≥ 10 ULN, AP ≥ 5 ULN, and/or bil ≥ 5 ULN as severe.

The data for these patients were analyzed using the SPSS Version 20 software. We used Parametric and nonparametric tests according to the nature of the variable and its distribution. *P* value less than 0.05 was considered statistically significant.

## Results

Our population was composed of 10 men and 7 women. The average age at admission was 41.4 ± 14.8 years (range 20–78 years). The average delay of hyperthyroidism diagnosis was estimated at 9.46 ± 8.5 months (range 2–36 months).

Four patients had a personal history of autoimmune diseases: type 1 diabetes and pernicious anemia in one and three patients, respectively.

The mean serum level of free thyroxine was 83.8 pmol/L (37.8–320). Serum TSH level was below the detection limit (≤0.02 mIU/L) in 10/17 cases. In the other seven cases, the mean serum level of TSH was 0.08 mUI/L (0.05–19). Thyroid peroxidase antibodies (TPO) (441.7 ± 263.9) and TSH receptor antibodies (TRAb) were positive in 13/14 and 5/6 cases, respectively.

Graves' disease (12/17) was the most frequently found etiology of hyperthyroidism, followed by Hashimoto's disease (4/17). Toxic multinodular goiter (TMG) was noted in one case.

The majority of our patients (14/17) had hyperthyroidism's complications. Cardiovascular consequences, thyrotoxic myopathy and hyperthyroid psychosis were noted in 13, 2 and 1 case, respectively. Eleven of 13 patients with cardiovascular complications had rhythm disturbance, mainly sinus tachycardia (6/12) followed by atrial fibrillation (5/12). Myocardial ischemia was noted in 4/13 patients. Eleven patients manifested congestive heart failure (CHF). A cardiac injury, aortic and mitral valvulopathy, was noted in one case. Only nine patients had a left ventricular ejection fraction (LVEF) measured by transthoracic echocardiography. It was depressed (<55%) in six cases.

For hepatic dysfunction, abdominal examination revealed ascites, splenomegaly, hepatomegaly, and hepato‐splenomegaly in 4, 2, 2, and 1 cases, respectively.

Liver biochemical tests are detailed in Table 1. We noted an elevation in serum levels of AST, ALT, AP, GGT, and Bil in 8, 7, 12, 12, and 10 cases, respectively. Hepatic dysfunction was mild, moderate, and severe in 7, 8, and 2 cases, respectively. The two patients with severe hepatic dysfunction had global heart failure.

**Table 1:** Mean, standard deviation, and range values of hepatic indices

	AST (U/L)	ALT (U/L)	GGT (U/L)	AP (U/L)	Bil (μmol/L)	Alb (g/L)	PR (%)
Median	39	35	61	313	24	31.5	61
Standard deviation	77.8	75.9	55.9	266.6	51	8.6	22.5
Range	20–347	2–250	17–232	54–1010	5.9–220	20–48	19–92

Alb, serum albumin; ALT, alanine aminotransferase; AP, alkaline phosphatase; AST, serum aspartate transaminotransferase; Bil, total bilirubin; GGT, gamma glutamyltransferase; PR, prothrombin ratio.

The LFT patterns noted in our patients were cholestasis, hepatocellular injury, and synthetic liver dysfunction. Fifteen patients had cholestasis, associated with jaundice in five cases. Hepatocellular injury and synthetic liver dysfunction were noted in seven and five cases, respectively. One patient had an isolated elevated bilirubin level. The three hepatic abnormalities were observed in two patients.

TPO was positively correlated with the serum level of bilirubin (*ρ* = 0.695; *P* = 0.038) (Table 2). A negative correlation was noted between ALT and LVEF (*ρ* = −0.812; *P* = 0.05). Benzylthiouracil was prescribed prior to radical approach for only three patients. No aggravation of hepatic parameters was noted. Radioactive iodine was indicated for the majority of our patients (15/17 cases) with doses ranging from 4.5 to 12 mCi (mean, 6.8 mCi). Total thyroidectomy was indicated in one case for a suspicious thyroid nodule. One patient died shortly after admission due to cardiovascular complications. Follow‐up liver tests were available in 11 cases. They all had normalized hepatic function once euthyroidism restored. Thus, a liver biopsy was not required.

**Table 2:** Correlation between thyroid, left ventricular ejection fraction and hepatic indices

	FT4	TPO	TRAb	LVEF
AST	*ρ* = 0.173	*ρ* = −0.317	*ρ* = −0.4	*ρ* = −0.754
	*P* = 0.506	*P* = 0.406	*P* = 0.6	*P* = 0.084
ALT	*ρ* = 0.255	*ρ* = −0.617	*ρ* = −0.4	***ρ* = −0.812**
	*P* = 0.324	*P* = 0.077	*P* = 0.6	***P* = 0.05**
AP	*ρ* = −0.41	r = 0.537	*ρ* = 0.8	*r* = −0.37
	*P* = 0.877	*P* = 0.136	*P* = 0.2	*P* = 0.471
GGT	*ρ* = −0.288	*ρ* = −0.571	*ρ* = −0.2	*ρ* = 0.588
	*P* = 0.262	*P* = 0.108	*P* = 0.8	*P* = 0.219
Bil	*ρ* = 0.269	***ρ* = 0.695**	*ρ* = −0.6	*ρ* = −0.29
	*P* = 0.297	***P* = 0.038**	*P* = 0.4	*P* = 0.577
PR	*ρ* = 0.009	*r* = −0.026	*ρ* = −0.5	*r* = 0.83
	*P* = 0.979	*P* = 0.96	*P* = 0.667	*P* = 0.17
Alb	*ρ* = −0.028	*r* = 0.148	*ρ* = 0.4	*r* = 0.742
	*P* = 0.929	*P* = 0.751	*P* = 0.6	*P* = 0.258

Bold values denote statistical significance at the *p* ≤ 0.05 level. Alb, serum albumin; ALT, alanine aminotransferase; AP, alkaline phosphatase; AST, serum aspartate transaminotransferase; Bil, total bilirubin; FT4, free thyroxine; GGT, gamma glutamyltransferase; LVEF, left ventricular ejection fraction; *P*, *P* value; PR, prothrombin ratio; TPO, thyroid peroxidase antibodies; TRAb, serum thyrotropin receptor antibodies; *r*, Pearson's correlation coefficient; *ρ*, Spearman's correlation coefficient.

## Discussion

Hepatic dysfunction is commonly observed in patients with thyroid disease. Thyroid hormones are glucuronidated and sulfated within the liver and subsequently excreted into bile, and also maintain the metabolism of bilirubin by regulating glucuronyl transferase and ligandin, a hepatic transport protein.^10^ Derangements in LFTs are common even in patients with subclinical hyperthyroidism but synthetic liver dysfunction is rare.^11^ A large number of series have reported the prevalence of liver test abnormalities, ranging between 15% and 76% of patients with hyperthyroidism.^12^, ^13^

In accordance with the literature,^14^, ^15^ increases in serum AP were followed in frequency by increases in levels of GGT, bilirubin, and aminotransferases. AP elevation, seen in 64% of patients with thyrotoxicosis,^16^ is due to increased osteoblastic activity with a predominant elevation of bone isoenzyme.^15^ Elevation of GGT levels is seen in 16.8–62% of hyperthyroid patients in various studies^17^, ^18^ and usually correlates well with AP.^17^ Cholestatis and synthetic liver dysfunction were the most and least noted presentation, respectively in our population. Some investigators have noted a pattern of cholestatic liver dysfunction, with case reports documenting severe jaundice as the predominant clinical feature.^19^

The etiology of liver injury in patients with hyperthyroidism covers a broad diagnostic spectrum. More than one cause may be identified at presentation.^14^ Specific risk factors for more profound liver disease in hyperthyroid patients remain incompletely understood. The mechanism for an association between thyroid hormone excess and hepatic dysfunction is unclear and may be due to indirect pathways or, alternatively, direct hormone effects on the target organ.^20^

Thyrotoxicosis may cause secondary hemodynamic dysfunction that can perturb liver function. CHF (with or without preexisting heart disease), often secondary to atrial fibrillation but also described in sinus tachycardia, has a recognized association with hyperthyroidism.^1^, ^15^ Studies of this association have found it to be an uncommon manifestation of hyperthyroidism generally.^21^ It also reduces LVEF in 3% of cases.^22^ Li *et al*.^23^ suggested that for the Graves' disease patients with the higher thyroid hormone of FT4 > 70.5 pmol/L and at the same time with the heart rate above 90 times per minute, the risk of hepatic function injury would increase. Fong *et al*.^12^ stated that indeed hepatic abnormalities were greater in the 19 patients with hyperthyroidism and CHF.

In our study, the only two patients with severe hepatic dysfunction had global heart failure.

Sherlock^24^ asserted that the cause of hepatic dysfunction is in fact CHF and that the liver histology is normal in hyperthyroid patients without heart failure.

CHF serves as a potential confounding variable as it is a well‐recognized cause of liver dysfunction. It usually induces mild alterations of LFTs. However, in cases of acute congestion, aminotransferase levels may reach values as high as those associated with viral or toxic hepatitis, and bilirubin may exceed 20 mg/dL, so it may act as a marker of disease severity. In our patient group, a negative correlation was noted between ALT and LVEF. A significant correlation between LVEF and the degree of hepatic abnormalities remains controversial.^1^, ^12^ It was suggested that the etiology of the deranged LFTs seen in patients with milder or subacute hyperthyroidism, as in our population, is more dependent on the longer‐term cardiac effects of excess thyroid hormone.^12^

No significant correlation between thyroxine and liver dysfunction was noted in our study, an observation that is in keeping with the results of previous reports.^12^, ^25^ However, a significant positive relationship between TPO and levels of bilirubin was observed (*ρ* = 0.695; *P* = 0.038). This suggests that there is more to hyperthyroidism effect on hepatic parameters than direct and/or indirect impact via hormones and that an autoimmune mechanism might be involved. In a recent study in China, Wang^26^ noted that a positive TPO was more likely to be associated with hepatic dysfunction (OR: 1.453–3.985, *P*  <  0.01).

Hepatic dysfunction is also attributed to the hypermetabolic state in thyrotoxicosis that increases hepatic oxygen consumption without increasing hepatic blood flow, accentuating the low oxygen tension in the centrilobular zones, possibly leading to dysfunction in the centrilobular hepatocytes.^27^ Histopathology of the liver is nonspecific (steatosis with or without fibrosis) and does not contribute to differential diagnosis.^12^, ^28^

The hepatic disorders are rapidly improved after the start of hyperthyroidism treatment and once euthyroid status is reached, as in our study.

A challenging decision for us was whether to use antithyroid agents knowing that they may cause hepatic complications. The estimated incidence of antithyroid associated hepatotoxicosis is estimated at 0.1–0.2%.^3^ Older age and higher dose of these drugs are risk factors for hepatic injury. Hepatic function should therefore be monitored routinely during treatment. Other options that have been successfully used in cases of antithyroid‐induced hepatotoxicity include substituting antithyroid agents, use of cholestyramine and radioiodine.^29^ Three patients in our study had antithyroid drug benzylthiouracil prior to radical approach, which consisted of radioactive iodine and total thyroidectomy. The metabolic process of 131I usually does not cause radiation damage to the liver. Therefore, 131I treatment should be the prior choice for patients with hepatic dysfunction in order to control hyperthyroidism timely and effectively.^26^

In conclusion, the clinical features of hyperthyroidism are diverse. Liver injury is relatively common, ranging from mild to severe. Therefore, patients presenting unexplained hepatic abnormalities require close examination and an evaluation of the thyroid function should be sought. The mechanism for such association is still unclear and may be due to indirect and/or direct pathways. Thus, further studies are needed for a better understanding of the etiopathogenesis and management of hepatic dysfunction in hyperthyroid patients.
